# Pyoderma gangrenosum in refractory celiac disease: a case report

**DOI:** 10.1186/1471-230X-13-162

**Published:** 2013-11-27

**Authors:** Silvia Sedda, Roberta Caruso, Irene Marafini, Elena Campione, Augusto Orlandi, Francesco Pallone, Giovanni Monteleone

**Affiliations:** 1 Department of Systems Medicine, University of Rome “Tor Vergata”, Via Montpellier, 1, 00133 Rome, Italy; 2 Department of Biomedicine and Prevention, Anatomic Pathology Unit, University of Rome “Tor Vergata”, Rome, Italy

**Keywords:** Gluten, Cutaneous ulcers, Celiac disease, Pyoderma gangrenosum

## Abstract

**Background:**

Pyoderma gangrenosum is an inflammatory neutrophilic dermatosis characterized by painful cutaneous ulcerations and often associated with systemic inflammatory and neoplastic diseases. Here we report the first case of pyoderma gangrenosum in a patient with refractory celiac disease.

**Case presentation:**

A 52-year-old woman with a previously diagnosed refractory celiac disease resistant to steroids and immunosuppressive drugs presented to our hospital for a rapidly growing, painful inflammatory skin lesion of the left leg. Physical examination revealed a painful lesion with focal ulceration, necrosis and pus discharge with active inflammatory borders at the external part of the left leg. Histological evaluation of a skin biopsy and analysis of inflammatory cytokines and matrix-degrading proteases in lesional skin samples confirmed the clinical suspicion of pyoderma gangrenosum. Treatment with oral prednisone was rapidly followed by a complete healing of the skin lesion but no improvement of symptoms/signs of malabsorption.

**Conclusion:**

Treatment of the patient with systemic steroids healed the skin lesion without improving the underlying refractory celiac disease. This observation raises the possibility that refractory celiac disease and pyoderma gangrenosum may be immunologically different.

## Background

Pyoderma gangrenosum (PG) is an inflammatory neutrophilic dermatosis characterized by painful cutaneous ulcerations persisting for more than 4 weeks [1]. Although accurate epidemiological data on PG are missing, the general incidence has been estimated to be between 3 and 10 per million per year, with a peak of incidence between the ages of 20 to 50 years [2-6]. Women are affected more frequently than men. Based on clinical presentation, PG can be differentiated in four major types: ulcerative, pustular, bullous and vegetative [3-5]. The diagnosis of PG requires the exclusion of other disorders, which can manifest with cutaneous ulceration (e.g. infections, vascular diseases, malignancies), and is based on clinical history, histopathological findings and response to therapy. Histopathological analysis of skin biopsies is useful to exclude other pathologies which clinically mimic PG rather than establishing a diagnosis of PG per se [3,4,7]. PG occurs most commonly on the lower legs with preference for the pretibial area or on peristomal areas. Other sites of involvement include breast, hand, trunk, head and neck. Moreover PG can have extracutaneous manifestations, such as involvement of upper airway mucosa, genital mucosa and eye, spleen infiltrates, neutrophilic myositis, sterile pulmonary neutrophilic infiltrates and sterile cortical osteolysis. It has been however suggested that the diagnosis of PG should be questioned when the legs or peristomal areas are not involved [3-5,7].

Although PG can manifest in individuals apparently healthy, it is frequently associated with inflammatory or neoplastic systemic diseases, such as inflammatory bowel diseases, rheumatic disorders, leukemia and myelodysplastic syndrome [3-5]. Furthermore, an association with such diseases in the context of skin ulcerations with crater-like holes and cribiform scarring helps make the final diagnosis of PG [3-5], thus highlighting the diagnostic relevance of identifying diseases which might be associated to PG.

Here we describe a case of PG complicating the natural history of a woman with refractory celiac disease (RCD), a form of celiac disease (CD) characterized by symptoms/signs of malabsorption and villous atrophy unresponsive to a strict gluten-free diet (GFD) [8].

## Case presentation

**Figure 1:**
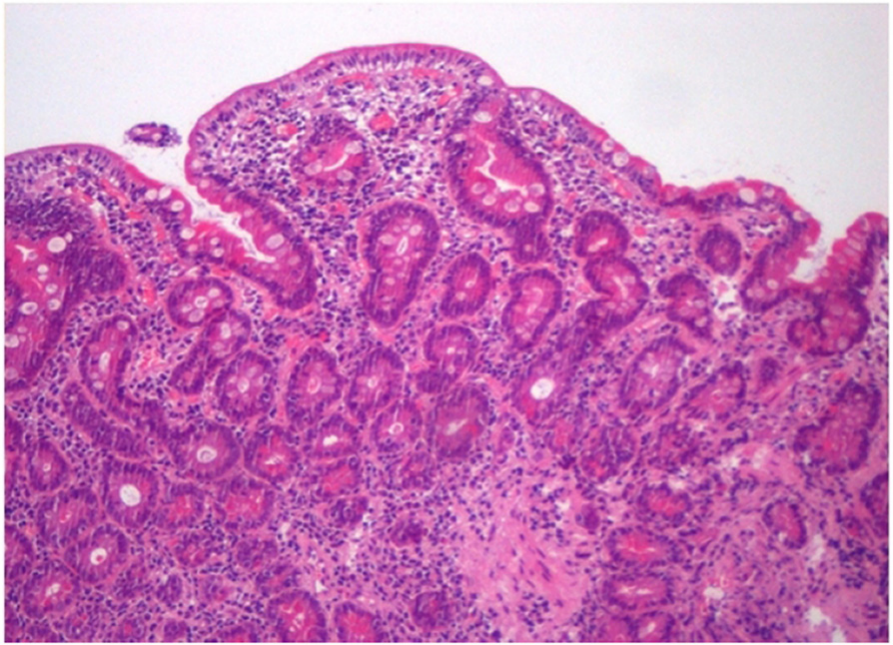
Photomicrographs of HE-stained paraffin section of a duodenal mucosal biopsy sample taken from a patient with refractory celiac disease showing subtotal villous atrophy and increased inflammatory cell infiltrates of the lamina propria compartment (original magnification 100x).

**Figure 2:**
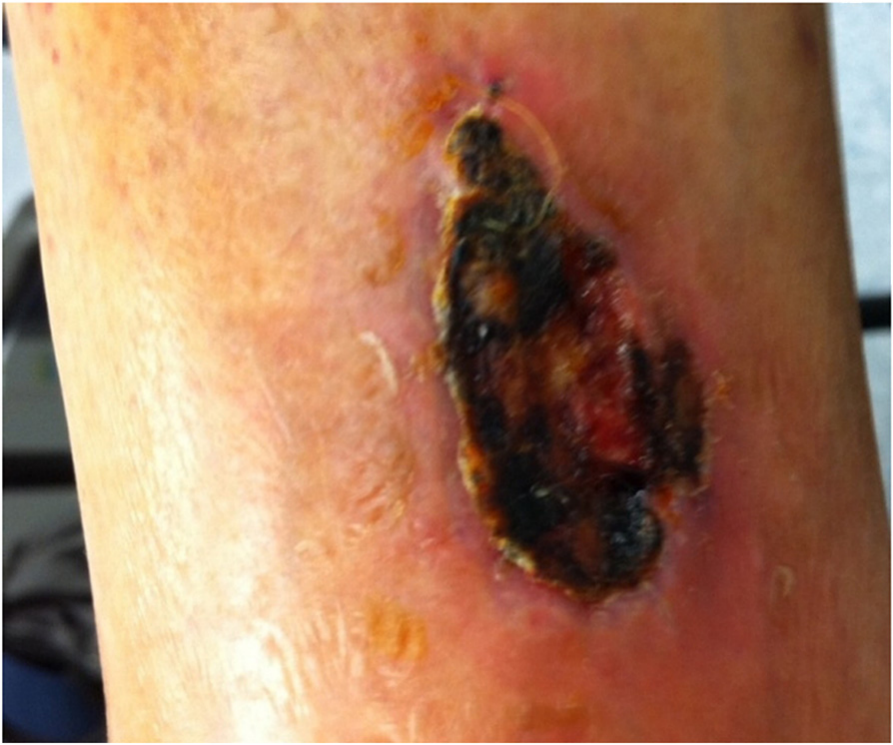
Ulcerative necrotic lesion on the anterior face of left leg.

A 52-year-old woman presented at the Gastroenterology Unit, University Tor Vergata Hospital, (Rome, Italy) in April 2011. Since 1990, the patient was complaining of diarrhea, abdominal pain and weight loss. In March 1993, CD was diagnosed based on positive serologic tests [anti-endomysium IgA antibody (EMA), anti-tissue transglutaminase-2 (TG-2) antibody], evidence of total villous atrophy of duodenal biopsies taken during upper gastrointestinal endoscopy, and positivity for human leukocyte antigen-DQ2. In October 2005, for the persistence of malabsorption symptoms/signs, despite a strict adherence to a GFD since 1993, the patient underwent further endoscopic and laboratory investigations, and eventually RCD was diagnosed. Therefore, she was treated with several courses of steroids and cyclosporine A with partial clinical response. Upon admission, she complained of abdominal pain, diarrhea (5 stools/day) and asthenia, despite she was on a strict GFD and treated with prednisolone (0.4 mg/kg/day). Her body mass index (BMI) was 15,6 kg/m^2^; the remaining physical examination was unremarkable. Laboratory analysis revealed exclusively anemia. Thyroid gland function tests were normal. EMA, anti-TG-2 and anti-enterocyte antibodies were negative. A bone density scan revealed low bone mineral density. Diagnostic work-up for infectious agents was negative. Colonoscopy with histological evaluation of colonic biopsies, CT enterography and small intestine contrast ultrasonography were normal. An upper endoscopy was performed and histological evaluation of duodenal biopsies showed an increased infiltration of the epithelial compartment with lymphocytes, crypts hyperplasia and a subtotal villous atrophy (Marsh 3b stage) (Figure 1). Further histological assessment of duodenal samples showed no collagen deposition. Diagnostic work-up for other villous atrophy-induced diseases (e.g. NSAIDs, olmesartan use, collagenous sprue and common variable immunodeficiency) was negative. Immunophenotyping analysis showed the absence of aberrant intraepithelial lymphocytes (RCD type I). Since a new course of steroids (prednisone 0.5 mg/kg/day) was unsuccessful and the patient refused to be treated with azathioprine, anti-TNF-α antibody infusion was scheduled for November 2011. However, in October 2011, she was reviewed again in our Hospital for a rapidly growing, painful inflammatory skin lesion of the left leg. At that time she was treated with oral prednisone (0.2 mg/kg/day) and complaining of diarrhea and abdominal pain. Physical examination revealed a painful lesion with focal ulceration, necrosis and pus discharge with active inflammatory borders at the external part of the left leg (Figure 2). Histological evaluation of a skin biopsy taken 7 days later in the Dermatology clinic at the Tor Vergata University Hospital, Rome, showed an irregular acantotic epidermis and underlying scarring dermis with acute and chronic inflammatory cell infiltrates. Additional biopsy samples were collected from both lesional and non-lesional skin and used to assess RNA expression of interleukin (IL)-8, IL-17A, matrix metalloproteinase (MMP)-2 and MMP-9, because these molecules are involved in the recruitment of neutrophils to the skin and supposed to play a key role in the pathogenesis of PG [9,10]. RNA transcripts of IL-17A, IL-8, MMP-2 and MMP-9 were more pronounced in the affected skin as compared to non-lesional skin (Table 1). PG was eventually diagnosed and patient was treated with oral prednisone (0.7 mg/kg/day). A dramatic improvement was observed; the pain diminished considerably and a complete healing of the skin lesion was documented in December 2011. No recurrence was observed after a follow-up period of 8 months.

**Table 1:** Cytokine and MMP transcripts in lesional and non-lesional skin of one refractory celiac disease patient

	**RNA transcript relative expression**			
	**IL-8**	**IL-17A**	**MMP-2**	**MMP-9**
Non-Lesional skin	1	1.2	1.1	1.1
Lesional skin	10.1	8.2	9	92.3

Total RNA was extracted using Pure Link mRNA mini kit according to the manufacturer’s instructions (Life Technologies, Milan, Italy). A constant amount of RNA (1 μg/sample) was retro-transcribed into complementary DNA (cDNA) and then 1 μl of cDNA/sample was amplified using the following conditions: denaturation 1 minute at 95°C; annealing 30 seconds at 55°C for MMP-2, at 58°C for IL-8 and MMP-9, at 61° for IL-17A and at 60°C for β-Actin, followed by 30 seconds of extension at 72°C. Primers’ sequences were as follows: IL-8 forward 5′-AGGAACCATCTCACTGTGTG-3′, reverse 5′-CCACTCTCAATCACTCTCAG-3′; IL-17A forward 5′-ACTACAACCGATCCACCTCAC-3′, reverse 5′-ACTTTGCCTCCCAGATCACAG-3′; MMP-2 forward 5′-TGACGGAAAGATGTGGTGTG-3′, reverse 5′-GGTGTAGGTGTAAATGGGTG-3′; MMP-9 forward 5′-CGTCTTCCAGTACCGAGAGA-3′, reverse 5′-GCAGGATGTCATAGGTCACG-3′; β-actin forward 5′-AAGATGACCCAGATCATGTTTGAGACC-3′, reverse 5′-AGCCAGTCCAGACGCAGGAT-3′ was used as internal control gene. RNA expression was calculated relative to the housekeeping β -actin gene on the base of the ΔΔCt algorithm.

*Abbreviations:* IL, interleukin, MMP, matrix metalloproteinase.

## Conclusions

CD is an immune-mediated enteropathy triggered by the ingestion of gluten in genetically-susceptible individuals [11]. The clinical spectrum of CD is wide, ranging from asymptomatic presentations to symptomatic cases with either classical intestinal (e.g. abdominal pain, chronic diarrhea, weight loss) or non-classical extraintestinal (e.g. anemia, osteoporosis) features [11]. CD can be associated with cutaneous manifestations, which can improve following exclusion of gluten from the diet. The most common CD-associated skin pathologies are dermatitis herpetiformis, which is characterized by itchy, chronic, papulo-vesicular eruption, and psoriasis [12].

This is the first case to show a possible association between RCD and PG. Interestingly, PG responded rapidly to steroids while no simultaneous improvement of the underlying RCD was seen. This raises the possibility that RCD and PG are immunologically different disorders. We feel it is fair to conclude that physicians should consider this putative association so that appropriate medical therapy can be started early on.

## Consent

“Written informed consent was obtained from the patient for publication of this Case report and any accompanying images. A copy of the written consent is available for review by the Editor of this journal”.

## Competing interests

‘The authors declare that they have no competing interests’.

## Authors’ contributions

S.S. collected clinical data; R.C. and I.M. performed real-time PCR; E.C. visited the patient in the dermatology unit; A.O. performed histological analysis of skin biopsies; F.P. and G.M. supervised the case and wrote the manuscript. All authors read and approved the final manuscript.

## Pre-publication history

The pre-publication history for this paper can be accessed here:

http://www.biomedcentral.com/1471-230X/13/162/prepub
